# Conic shapes have higher sensitivity than cylindrical ones in nanopore DNA sequencing

**DOI:** 10.1038/s41598-018-27517-8

**Published:** 2018-06-14

**Authors:** Bin Tu, Shiyang Bai, Benzhuo Lu, Qiaojun Fang

**Affiliations:** 1 CAS Key Laboratory for Biomedical Effects of Nanomaterials and Nanosafety, CAS Center for Excellence in Nanoscience, National Center for Nanoscience and Technology, beijing, 100190 China; 2 Academy of Mathematics and Systems Science, Chinese Academy of Sciences, beijing, 100190 China; 3 Beijing Key Laboratory of Ambient Particles Health Effects and Prevention Techniques, beijing, 100190 China; 4 University of Chinese Academy of Sciences, Beijing, 100049 China; 5 Sino-Danish Center for Education and Research, Beijing, 101408 China

## Abstract

Nanopores have emerged as helpful research tools for single molecule detection. Through continuum modeling, we investigated the effects of membrane thickness, nanopore size, and pore shape on current signal characteristics of DNA. The simulation results showed that, when reducing the pore diameter, the amplitudes of current signals of DNA increase. Moreover, we found that, compared to cylindrically shaped nanopores, conical-shaped nanopores produce greater signal amplitudes from biomolecules translocation. Finally, we demonstrated that continuum model simulations for the discrimination of DNA and RNA yield current characteristics approximately consistent with experimental measurements and that A-T and G-C base pairs can be distinguished using thin conical solid-state nanopores. Our study not only suggests that computational approaches in this work can be used to guide the designs of nanopore for single molecule detection, but it also provides several possible ways to improve the current amplitudes of nanopores for better resolution.

## Introduction

Nanopores have emerged as promising next generation devices for DNA sequencing. Biomolecules passing through a nanopore yield an ionic current trace, which provides information of the physical and chemical properties of the Biomolecule. The sequential translocation of four nucleotides of DNA, will yield an distinct ionic current characteristic. The nanopore technique has been applied to characterize and analyze all kinds of biomolecules^1–4^.

In the initial concept of nanopore sequencing, an individual single stranded DNA (ssDNA) molecule was threaded through the *α*-haemolysin (*α*HL) protein pore^5^. Subsequently, two other biological nanopores, MspA^6^ and phi29 connector^7^, were used to investigate ssDNA/dsDNA sequencing. Two different approaches to DNA sequencing were shown with biological protein nanopores directly reading of the four bases^8,9^. Recently, a promising wild-type aerolysin nanopore was used to resolve individual short oligonucleotides^10^. Biological nanopores have shown great potential for the construction of nanopore sequencing devices, which have proven useful for separating polymers at high resolution. Yet, the system still suffers typically from limitations, including flaw to external vibrations, such as PH, temperature, and mechanical oscillations^2^. The phi29 connector was chemically stable over a range of pH, temperature and ionic strength^11,12^.

Solid-state nanopores have emerged as an promising alternative to biological pores, which have shown to be chemically stable over a range of PH and temperature^1^. Extensive studies have been performed on ssDNA translocation^13,14^, dsDNA translocation^15–20^, and protein translocation^21,22^ through solid-state nanopores. Solid-state nanopore are less reproducible and easily engineered for specific nanopore modification or conjugation. Solid-state nanopores are usually dozens of nanometers thick, making it difficult to detect individual base by observing the ionic current as multiple bases interact with the nanopore simultaneously, which have currently not been able alone to characterize ssDNA^23^.

In the case of solid-state nanopores, DNA translocation can be affected by many factors, such as the temperature, electrolyte viscosity, ion concentration, pore diameter, and membrane thickness. A common problem is that the speed of biomolecules transpassing through the nanopores is too high to detect the local structure via ionic current measurement. Various experimental and computational approaches approaches have been applied to slow the speed of biomolecules translocating through nanopores^24,25^, such as temperature^26^, optical tweezers^27^ and modifications to the solvent ^18,28^. Another problem is that single-base resolution of the current is not high enough for some kinds of real-time sequencing. An possibly way to solve this problem is to increase the signal amplitudes. The membrane thickness has already been investigated to increase the signal amplitudes for improving the resolution of the current reading. Wanunu *et al*.^29^ discovered that reducing the thickness of the SiN nanopore led to increased signal amplitudes from biomolecules.

Molecular dynamics (MD) simulations have been used to study the ionic current flow and single molecule transport through protein and solid-state nanopores^30–33^. Using Atomic-resolution Brownian dynamics (ARBD) method, researchers found that a small change in the sequence of DNA within a pore can cause a large change in the ion current^34^. PNP theory has been previously applied to the study of ion current rectification in charged conical nanopores^35^, electron transport in semiconductor devices^36^, and ion permeation through biological membrane channels^37–42^. These has a few shortcomings regarding the applicability of the PNP theory in the nanoscale systems. The PNP model neglects the finite volume effect of ion particles, which is especially important for narrow nanopores^43^. Actually, a modified PNP model that explicitly takes into account ion exclusion has been previously applied to determine ion flow through a nanopore^44^. Additionally, non-electrostatic interactions between ions are excluded in the PNP model. Finally, there is a lack of the description of dielectric boundary effects in the PNP theory^45^. Nevertheless, the PNP theory incorporates realistic channel structure and discrete protein charge locations in its modeling and computation, and treats the electrostatic potential distribution, concentration and flux of ions in the system in a self-consistent manner. Consequently, the PNP theory works well for wide ion nanopore (the diameter of pore 1 nm) transport at a relatively small computational cost^46^.

In this paper, 3D Poisson-Nernst-Planck (PNP) simulations were carried out to determine current signal characteristics of the presence of DNA/RNA within conical and cylinder SiN nanopores. Using PNP simulations, we first investigated the effect of geometrical features of SiN nanopores on the signal amplitudes from DNA, including pore size, membrane thickness, and pore shape. We showed that reducing the thickness of the SiN membrane led to increased signal amplitudes from DNA, consistent with experimental observations^29^. Previous studies have indicated that narrowing the pore size could slow the translocation of DNA through solid-state nanopores and give a better resolution to resolve different lengths of DNA. However, we investigated the effect of pore diameter on the current signal under different bias potentials, and found that reducing the pore diameter led to increased signal amplitudes from DNA. Moreover, We showed that, compared with cylindrically shaped nanopores, cone-shaped nanopores can improve the signal amplitudes from biomolecules. Then, based on the factors influencing the signal amplitude, we investigated the possibility of distinguishing A-T and G-C base pairs with an optimal thin conical nanopore.

## Results

**Figure 1:**
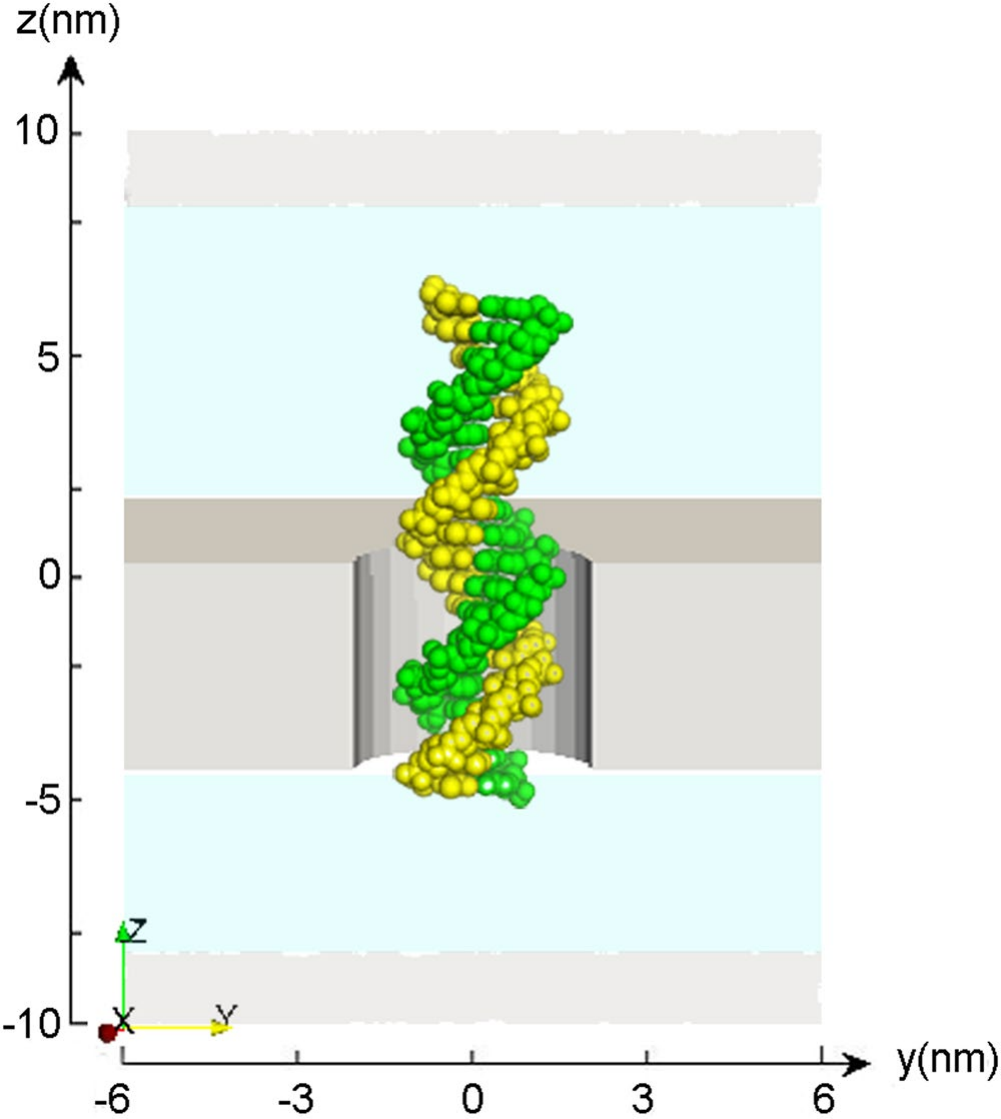
Schematics of the cylinder solid-state nanopore system. The system comprising DNA and SiN was set in the center of a simulation box. Shown is dsDNA statically put in a 4 nm-diameter nanopore. The SiN membrane is shown in gray.

Figure 1 illustrates a solid-state nanopore system consisting of a nanopore with SiN membrane and DNA. We performed continuum model simulations to obtain the current signals of DNA/RNA translocation through SiN nanopores. The simulations characterize the effects of pore size, membrane thickness, and pore shape on ionic current signals. In all of the following PNP simulations, the membrane thickness *h* is equal to the effective membrane thickness *h*_eff_^29^.

### The Effectiveness of 3D PNP Simulation of Open Pore Conductance

We first investigated how open pore characteristics of various diameters and membrane thicknesses affect ion current, which is crucial for computational study of DNA translocation-induced blockades in ionic current through nanopores. In the system comprising DNA and SiN shown in Fig. 1, anions and cations are driven in opposite directions by an external electric field, resulting in a net current. The simulation was performed to predict the ionic current through open pores at 1 M KCl solution with different pore diameters and membrane thicknesses. Detailed parameters are described in the methods.

**Figure 2:**
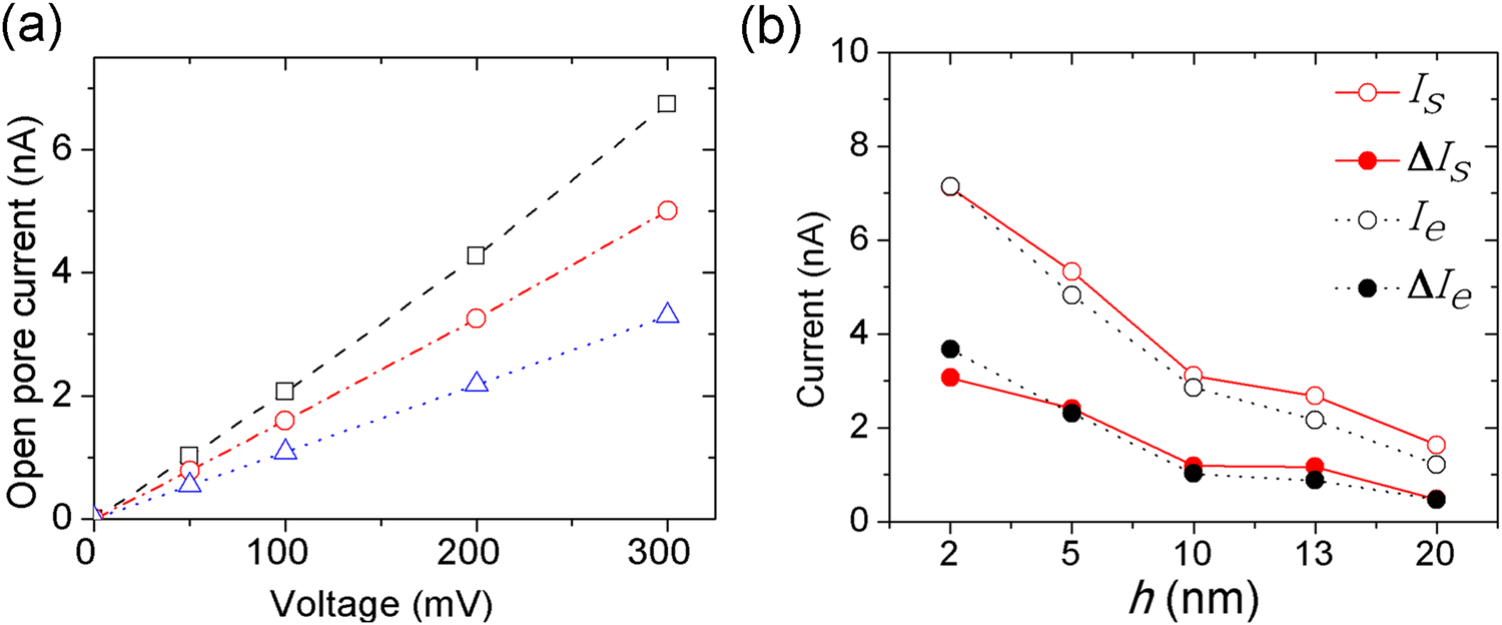
(**a**) I–V curves for three pores were obtained from the PNP calculations with 1 M KCl and a temperature of 21 °C, with the diameter and membrane thickness (*d*, *h*) = (4, 2.7) (black open squares), (4, 5) (red open circles) and (4, 8) (blue open triangles). (**b**) Dependence of open-pore currents and current amplitudes on *h*. Plots for comparisons between the experimental open-pore currents *I*_*e*_ (black open circles) and the simulation open-pore currents *I*_*s*_ (red open circles), the experimental current amplitudes Δ*I*_*e*_ (black solid circles) and the simulation current amplitudes Δ*I*_*s*_ (red solid circles). On decreasing h from 20 to 2 nm, the open-pore current increased, and the DNA signal amplitude increased.

To assess the accuracy of the continuum model simulation, results from PNP calculation were compared with the conductance of nanopores obtained from simulations with experimental results. Figure 2a shows current-voltage (I–V) curves for 4 nm-diameter pores with different membrane thicknesses under a bias voltage within the range 0–300 mV. We observed a linear I–V curve with the low applied bias voltages from 0 to 300 mV, similar to the trends in I–V curves observed with graphene nanopores^47^. In the meantime, we found that at the same applied bias voltage, the open-pore current increases as the membrane thickness *h* decreases from 8 to 2.7 nm, which is consistent with Wanunu *et al*.^29^.

The ionic conductance can be obtained as follows: $G=\frac{\delta I}{\delta V}$. We used the experimental conductance obtained from Wanunu *et al*.^29^ as the reference data for comparison. Table 1 shows the comparisons of the conductance between the simulation results and the experimental data. It can be seen that there is a good agreement between the two sets of data with a small deviation.

**Table 1:** The comparison between the simulation results (G_*sim*_) and experimental data (G_*exp*_).

(*d*, *h*)	Simulation results (nS)	Experimental data (nS)	Relative error
(6,2.7)	43.11	43.61	1.2%
(4,2.7)	24.63	24.89	1.1%
(4,5)	16.51	16.01	3.0%
(3,2.3)	14.12	13.80	2.3%
(4,8)	10.31	9.40	8.8%
(4,16.7)	4.01	3.92	2.2%

Relative error can be calculated by |(*G*_*sim*_ − *G*_*exp*_)/G_*sim*_|.

### Influence of membrane thickness, pore diameter, and shape on current signal

To determine the effect of membrane thickness on the signal amplitudes from dsDNA, a number of 4 nm-diameter pores with membrane thickness *h* = 2–20 nm were constructed. To ensure that the distance between the boundary of boxes and the mouth of pores unchanged, the size of simulation boxes were adaptively adjusted with the membrane thickness. In our simulations, a 20-nucleotide fragment of dsDNA was threaded through a nanopore in an SiN membrane, submerged in 1 M KCl solution. We performed PNP simulations for dsDNA molecules transporting through 4 nm-diameter pores of different thicknesses under the condition 21 °C and 300 mV. Figure 2b shows plots of the experimental open-pore currents *I*_*e*_ (black triangles), the simulation open-pore currents *I*_*s*_ (red triangles), the experimental current amplitudes Δ*I*_*e*_ (black circles), and the simulation current amplitudes Δ*I*_*s*_ (red circles). We found that, when decreasing the membrane thickness *h*, open pore currents increase and amplitudes of the current signals increase, which agrees well with the experimental observations^29^.

**Figure 3:**
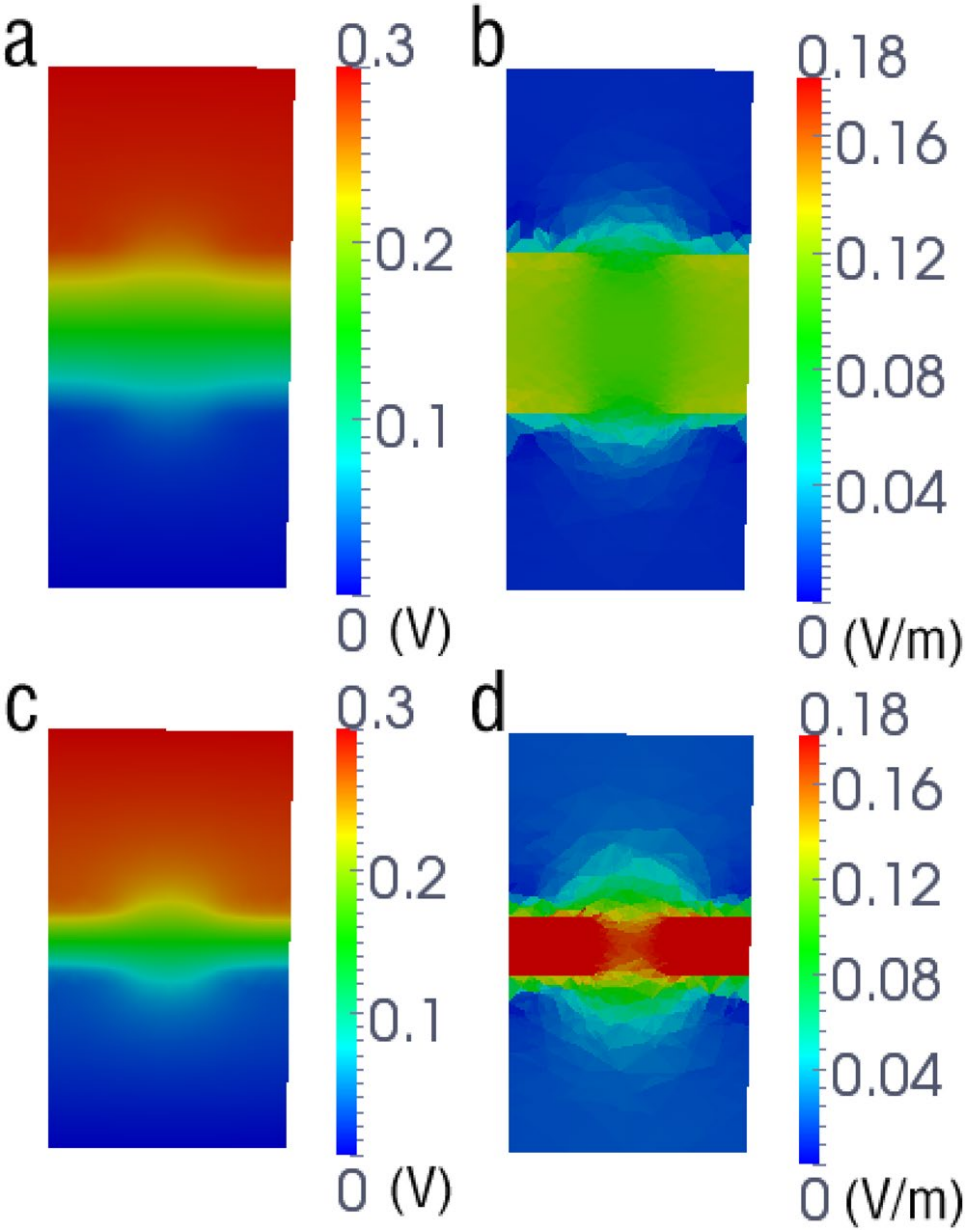
Shown are the electrostatic potential (V) and the magnitude of electric field intensity (V/m) maps in the (x,z) plane for membrane thicknesses of (**a**,**b**) 8 and (**c**,**d**) 3 nm respectively. The electrostatic potential and the magnitude of electric field intensity were obtained from PNP calculations with 1 M KCl and 300 mV.

To better understand the dependence of current signals on membrane thickness, the mean electrostatic potential and electric field intensity in the system were calculated. Figure 3 shows the electrostatic potential and electric field intensity maps in the (x,z)-plane for membrane thicknesses of 3 and 8 nm, respectively. The potential maps (Fig. 3a and c) illustrate that the electrostatic potential changes rapidly across the membrane. As shown in Fig. 3b and d, the magnitude of the electric field intensity in the pore region is ~0.1 V/m for 8 nm, and it is 0.16 ~ V/m for 3 nm.

**Figure 4:**
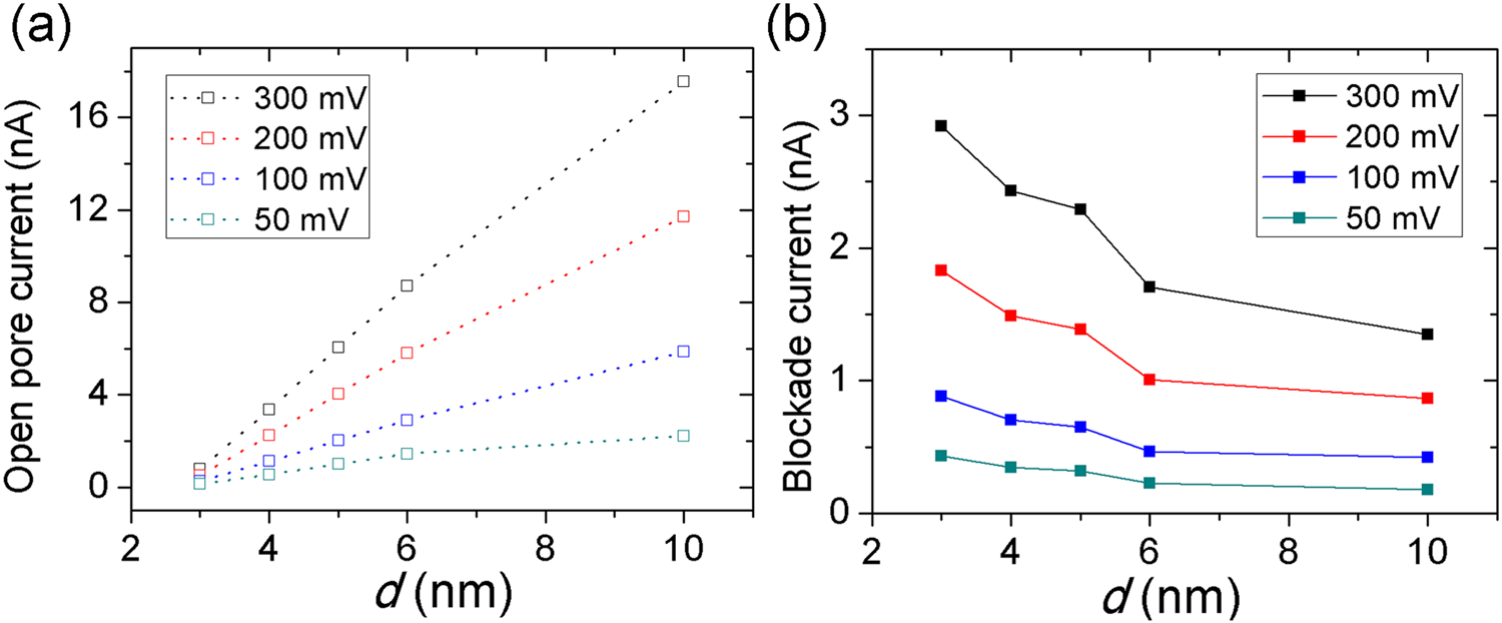
Dependence of open-pore currents *I*_*s*_ and current amplitudes Δ*I*_*s*_ on *d* (3–10 nm). The ionic currents were obtained from PNP simulations at 50 mV, 100 mV, 200 mV, and 300 mV bias voltages and 1 M KCl with presence of 20-bp dsDNA in five different nanopore diameters (3 nm, 4 nm, 5 nm, 6 nm, and 10 nm). When increasing *d* from 3 to 10 nm, open pore currents *I*_*s*_ increased (**a**); however, the DNA signal amplitude Δ*I*_*s*_ decreased (**b**).

In this study, using PNP simulations, we investigated the influence of pore diameter on the current signal of dsDNA molecules; PNP simulations were carried out for translocations of 20-bp dsDNA through *d* = 3–10 nm nanopores of *h* = 4 nm. Current values were obtained by solving 3d PNP equations with 50–300 mV and an electrolyte temperature of 21 °C. Figure 4a shows, when on increasing *d* from 3 to 10 nm, open pore currents *I*_*s*_ increase. However, as shown in Fig. 4b, the DNA signal amplitude Δ*I*_*s*_ decreased.

**Figure 5:**
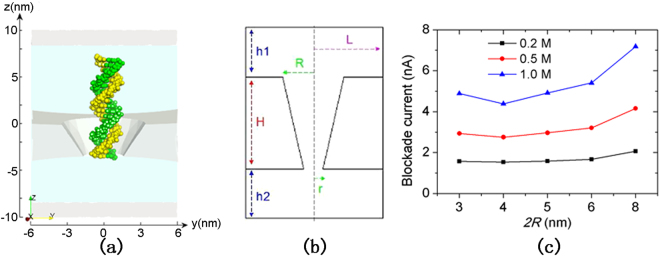
Conical nanopores. (**a**) Schematics of the conical nanopore system. (**b**) A 2D view of the geometry model of the conical nanopore system. All parameters shown in the figure can be adjusted for PNP simulations with various nanopore shapes. (**c**) Dependence of current signals on pore geometry (2*r* = 4 nm, 2*R* = 3 nm−8 nm, and *h* = 4 nm). The current amplitudes Δ*I*_*s*_ were obtained from PNP simulations of 20-bp dsDNA performed on those conical nanopores with a bias voltage of 300 mV and ion concentrations of 0.2 (black square), 0.5 (red circle), and 1.0 M (blue triangle) KCl, respectively.

To investigate the effect of pore shape on the signal amplitudes from dsDNA molecules, we constructed a conical nanopore system, as shown in Fig. 5a. Conical nanopores with various slopes were constructed for the comparison. We set the membrane thickness of the pore (*h*) at 4 nm; while the bottom diameter (2*r*) at 4 nm, the top diameter (2*R*) ranged from 3 to 8 nm, as shown in Fig. 5b. It should be noted that when 2*R* is equal to the same value as 2*r*, the conical nanopore becomes a cylinder nanopore. PNP simulations on these conical systems with a bias voltage of 300 mV and 1 M KCl concentrations at 0.2, 0.5, and 1.0 M were performed. Figure 5c shows that, when increasing 2*R* from 3 to 8 nm, the signal amplitude of dsDNA molecules is at a minimum of 2*R* = 4 nm (i.e., 2*R* = 2*r*). Furthermore, it increases as the difference between *R* and *r* increases (the maximum signal amplitude is 2*R* = 8 nm). This indicates that, compared with cylindrically shaped nanopores, nanopores in a conical shape can improve amplitudes of current signals in presence of DNA within nanopore.

### Discrimination between DNA and RNA

we first tested a cylinder thin pore (2*r* = 2*R* = 3 nm, *h* = 3 nm) to discriminate between DNA and RNA, then to test the ability of the conical nanopore for the discrimination between A-T and G-C base pairs. We used 22-bp RNA and 25-bp DNA for the simulations because the two molecules have the similar length; however they also have different cross-sectional areas due to their different helicities (PDB ID: 1RPU and 2BNA). PNP equations were calculated under 500 mV, 1 M KCl, and an electrolyte temperature of 21 °C. Using PNP simulations, we obtained the simulated current amplitudes of Δ*I*_*DNA*_ = 1.95 nA and Δ*I*_*RNA*_ = 2.76 nA, with the difference between 22-bp DNA and 22-bp RNA ~30%, which is in good agreement with their cross-sectional area differences^48^.

**Figure 6:**
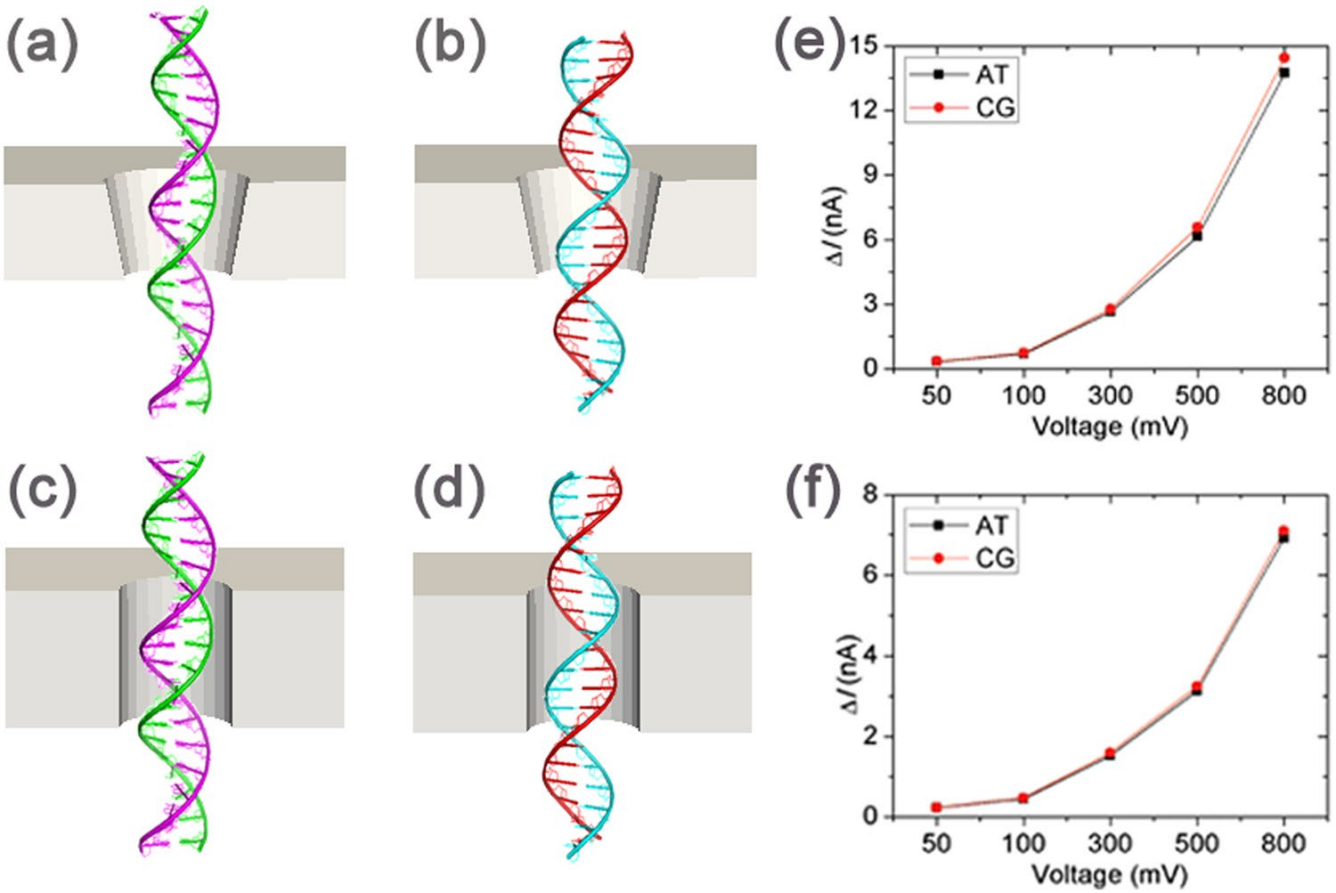
Discrimination between A-T and G-C base pairs. Schematics of conical nanopore (2*r* = 4 nm, 2*R* = 3 nm, *h* = 2.3 nm) with 25-bp AT (**a**) and 25-bp CG (**b**); And cylinder nanopore (2*r* = 4 nm, 2*R* = 4 nm, *h* = 5 nm) with 25-bp AT (**c**) and 25-bp CG (**d**); (**e**) and (**f**) are the current amplitude Δ*I* for the conical nanopore system and cylinder nanopore system respectively; At the same bias voltage, the difference between the current amplitude of 25-bp CG and 25-bp AT for the conical nanopore system is larger than that for the cylinder nanopore system.

Based on the above factors hta were analyzed to affect signal amplitude, we constructed a thin conical nanopore (2*r* = 4 nm, 2*R* = 3 nm, *h* = 2.3 nm) and a common cylinder nanopore (2*r* = 4 nm, 2*R* = 4 nm, *h* = 5 nm) to discriminate between A-T and G-C base pairs. PNP simulations were carried out for translocations of 25-bp AT and 25-bp CG through two nanopores with five bias voltages (50 mV, 100 mV, 300 mV, 500 mV and 800 mV). Figure 6f shows that the current amplitudes obtained were similar for 25-bp AT and 25-bp CG with the cylinder nanopore. For the conical nanopore (as shown in Fig. 6e) at a low bias potential (<300 mV), the current amplitudes of 25-bp AT and 25-bp CG are close, while at larger bias values from 500 mV to 800 mV, the current amplitude of 25-bp CG is larger than that of 25-bp AT. At a 500 mV bias voltage, the difference between the current amplitude of 25-bp CG (Δ*I*_*CG*_ = 6.58 nA) and 25-bp AT (Δ*I*_*AT*_ = 6.14 nA) is 440 pA for the conical nanopore; for the cylinder nanopore, it is 170 pA (Δ*I*_*CG*_ = 2.99 nA and Δ*I*_*AT*_ = 2.82 nA).

## Discussion

DNA sequencing has been known as one of the most prominent novel applications for nanopores, which is achieved through DNA translocation-induced current signal amplitudes. Increasing the signal amplitudes of biomolecules is helpful for the improvement of the accuracy of DNA sequencing. we have demonstrated the effect of pore size, membrane thickness, and pore shape on the current signal characteristics of DNA/RNA translocation.

We employed 3D PNP calculations to determine the steady-state ion conduction characteristics of solid-state nanopores. Our results indicate that a 3D PNP simulation can accurately reflect ion current through a nanopore and thus can effectively model open nanopore conductance. The 3D PNP model was also shown to be effective by the consistent experimental observations that on decreasing the membrane thickness *h*, the electric field strengthens, and thus leads to the increase of open pore currents and amplitudes of the current signals^29^.

Another important conclusion concerns the effect of the pore size on ionic current signals, which is expected to resolve the DNA/RNA sequence^47^. Previous studies have reported that a smaller pore gives better resolution to resolve different lengths of DNA^49^, and that decreasing the nanopore diameter can slow the speed of single-stranded DNA translocation through a solid-state nanopore^50^. By simulation of current signal of presence of DNA within the nanopore with diameters from 3 to 10 nm, we have shown that nanopores with smaller diameters led to the increased signal amplitudes. The underlying reason is obvious because when DNA translocates into the pore region, the percentage of the blocked area of the total cross-sectional area is higher for a smaller nanopore than for a bigger pore. Actually, previous experimental studies reported that the ratio of measured conductance of the phi29 connector to a-HL biological nanopore compares well with the ratio of cross-sectional areas, because the conductance of a channel is proportional to its cross-sectional area^7^. Moreover, by using atomic-resolution Brownian dynamics method, previous simulation studies reported that decreasing the cross section of the solid nanopore led to an decreased blocked current^51^. Therefore, nanopores with smaller diameters will result in larger signal amplitudes from dsDNA molecules.

Additionally, the effect of pore shape on the signal amplitudes from dsDNA molecules was investigated. Our study suggests that, when increasing the difference between the diameter of the top and bottom pore, the amplitudes of the current signals increase. It is probably because, when increasing the difference between *R* and *r*, the the narrow region decreases. Actually, the characteristics of the ionic current blockades are determined primarily by the polynucleotide segment threaded through the narrow region of the pore^52^. Previous studies reported that the MspA nanopore may provide improved spatial resolution, which has a shorter narrow region than the *α*-HL channel^6^. The phi29 connector is a rare conic shaped biological nanopre in previous experimental studies, which has been used successfully for single molecule detection and produces higher current signal amplitude in comparing to other biological pores^7,53^. Therefore, the bigger the difference between *R* and *r* is, the larger the ionic current and current amplitude are.

Finally, we performed PNP simulations for the discrimination of DNA and RNA, and the current signal characteristics obtained by theoretical calculations are consistent with experimental measurements^48^. We demonstrates that A-T and G-C base pairs can be better distinguished using conical SiN nanopores than cylinder ones at an appropriate voltage bias.

In summary, our PNP simulations successfully modeled the signal amplitudes from DNA/RNA translocating nanopores and how to improve the signals by altering the geometrical features of SiN nanopores (e.g. pore size, membrane thickness and pore shape). Although the PNP theory often forsake a good few of the biomolecular details, it can be seized of the advantage of superior computational efficiency and thus the calculations for long time scales are quite feasible^46^. We provided a connection between experimental data and simulation work to study the factors influencing the current signals from the presence of DNA/RNA within a solid-state nanopore.

## Methods

### Poisson-Nernst-Planck theory

In the PNP theory, ion flux includes a Fickian diffusion term and a drift term to account for the effects of the electrostatic field:

1 $$\frac{\partial {n}_{i}}{\partial t}=\nabla \cdot ({D}_{i}(\nabla {n}_{i}+\frac{{q}_{i}e}{{k}_{B}T}{n}_{i}\nabla u)),$$

where the index *i* corresponds to each of the N diffusing ionic species. *u* is the electrostatic potential, *n*_*i*_ is the concentration of species *i*, *D*_*i*_ is the (space-dependent) diffusion coefficient of species *i*, *q*_*i*_ is the charge of species *i e*., the elementary charge, *k*_*B*_ is Boltzmann’s constant, and *T* is the temperature, set to 298 *K* for all cases.

The electrostatic potential field, *u*, depends on any fixed charges arising from the protein channel as well as the mobile charge arising from the space-dependent ion concentrations through the Poisson equation:

2 $$-\nabla \cdot (\varepsilon \nabla u)-\sum _{i}{q}_{i}{n}_{i}=\sum _{i}{\rm{\Delta }}(x-{x}_{i}),$$

Here, *ε* is the (space-dependent) dielectric constant.

In the present work, the coupled Eqs (1) and (2) were solved numerically to obtain ion concentrations and electrostatic potential. The open-pore current (*I*_*o*_) and blocked DNA current (*I*_*DNA*_) can be obtained by the using following formula:

3 $$I=-\sum _{i}{q}_{i}{\int }_{S}({D}_{i}\frac{\partial {n}_{i}}{\partial z}+\frac{{q}_{i}{D}_{i}}{{k}_{B}T}{n}_{i}\frac{\partial u}{\partial z})dxdy\mathrm{.}$$

where *S* is a cut plane at any cross section inside the pore. Eq. (3) can be applied at any *z*-position along the pore axis, and it shows only minor differences in the current values *I* due to numerical inaccuracies. After obtaining open-pore currents and blocked DNA currents, the current amplitudes of DNA signals (Δ*I*) can be calculated as:

4 $${\rm{\Delta }}I={I}_{o}-{I}_{DNA}$$

The coupled PNP system (Eqs (1) and (2)) is solved on a 3D domain defined by the nanopore geometry. The voltage applied to the system, *V*_applied_, is given by the potential difference along the *z* direction. On the domain side boundary faces, the potential is set by interpolating linearly between top and bottom potential values. Ion concentrations on the top and bottom side boundaries are set to their bulk values. Additionally, there is a no-flux boundary surrounding the DNA/RNA and membrane that prevents ions from penetrating through the region occupied by the biomolecules and membrane.

### System setup

The model system consists of a nanopore with an SiN membrane and biomolecules. To numerically model the system, a simulation box containing the nanopore and membrane is required. The system comprising DNA and SiN was set in the center of a simulation box. a DNA/RNA molecule was statically put in the solid-state nanopore, which is aligned along the z axis. The size of the simulation box can be adaptively changed with the size of the nanopore and DNA.

A single/double-stranded helix of DNA/RNA was built with the program X3DNA^54^. The partial charges and atomic radii for each atom in the DNA/RNA were obtained by using the PDB2PQR^55^ software. The dielectric values of *ε* = 2 and *ε* = 78 were assigned to membrane and DNA regions as well as solvent regions, respectively. In the bulk region, the diffusion coefficients of the cations and anions were set to their experimental values: *D*_Cl_ = 0.203 Å^2^/*ps*, *D*_*K*_ = 0.196 Å^2^/*ps*. Generally, the diffusion coefficients in the bulk region and the channel pore region should be different, particularly for narrow pores^56^. In the pore region, we used the following correlation to calculate the diffusion coefficient of ions as a function of the pore radius^57^:

5 $${D}_{i,p}(r)=\frac{{D}_{i,b}}{A+Bexp(\beta /C)+Dexp(\beta /E)}$$

where *β* = *R*_*i*_/*R*_*p*_, and the empirical fitting parameters have the values given in Noskov *et al*. (A = 0.64309, B = 0.00044, C = 0.06894, D = 0.35647, E = 0.19409). The two ion radii *R*_*i*_ used in our model were the van der Waals radii specified in the CHARMM27 force field (*R*_*Cl*−_ = 2.27 A, *R*_*K* +_  = 1.764 A), and the radius of the pore *R*_*p*_ denotes the radius of the nanopore in the presence of a DNA molecule, which was calculated by *R*_*p*_ = *R* − *sqrt*(*S*/*π*). *R* denotes the radius of the open pore without a DNA/RNA molecule, and *S* denotes the cross sectional area of the biomolecule in the *XY*-plane. We used the following piecewise continuous function for the diffusion coefficient to make the diffusion coefficient of ions continuously change through the whole domain^37,58^.

6 $$D(r)=\{\begin{array}{ll}{D}_{{\rm{ion}}}, & r\in {\rm{bulkregion}},\\ {D}_{{\rm{chan}}}+({D}_{{\rm{chan}}}-{D}_{{\rm{ion}}})f(r), & r\in {\rm{bufferingregion}},\\ {D}_{{\rm{chan}}}, & r\in {\rm{channelregion}},\end{array}$$

where the function *f*(*r*) is given by

7 $$f(r)=f(z)=n{(\frac{z-{z}_{{\rm{chan}}}}{{z}_{{\rm{ion}}}-{z}_{{\rm{chan}}}})}^{n+1}-(n+\mathrm{1)}{(\frac{z-{z}_{{\rm{chan}}}}{{z}_{{\rm{ion}}}-{z}_{{\rm{chan}}}})}^{n},$$

where *n* is an integer and we set *n* = 7 in our computations. *z*_chan_ is the boundary value of channel region on *z* axis and *z*_ion_ is the boundary value of bulk region on *z* axis.

**Figure 7:**
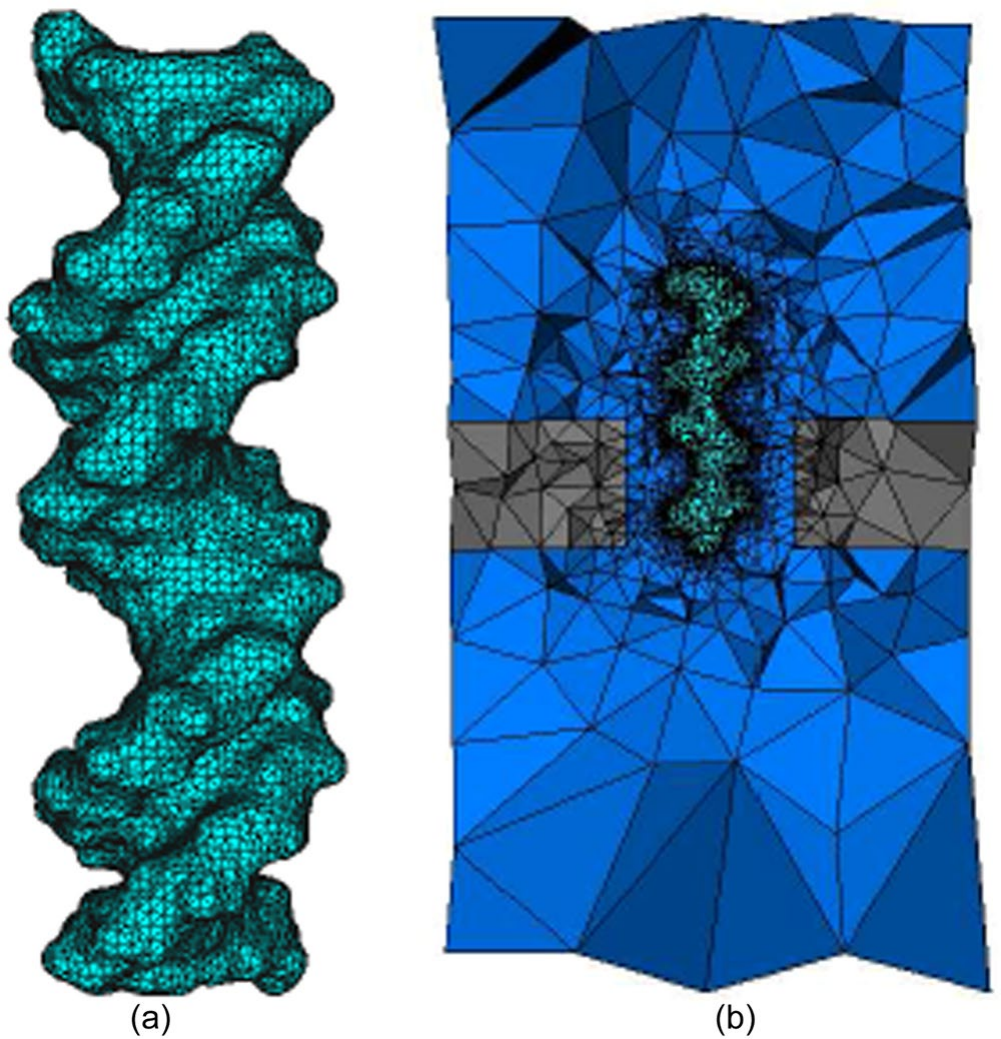
Mesh generation for a solid-state nanopore system. (**a**) Triangular boundary mesh conforming to DNA surface; (**b**) a view of a cross section of the whole tetrahedral volume mesh.

In our previous work, a tool chain was built for high-quality biomolecule volume mesh generation^37^. In this work, the meshes for solid-state nanopore systems are generated by using this tool chain. Figure 7a shows a triangular surface mesh of the 20-bp DNA molecule. An unstructured tetrahedral volume mesh of a solid-state nanopore system is shown in Fig. 7b.

### Stabilized Finite Element methods

Two stabilized method, the Pseudo Residual-Free Bubble (PRFB) method and the SUPG stablized method^44^, were applied to solve PNP equations in our simulations. Here, we briefly present the SUPG method to solve the NP equations. More details of PRFB method can be found in our previous work^59^. The stabilized weak form of (1) is approximated as follows:

For each *i*, to find ${n}_{i}\in {H}_{a}^{1}({\rm{\Omega }})$ which satisfies

8 $$G({n}_{i},\,v)+Q({n}_{i},\,{v}_{s})=\mathrm{0,}$$

where

9 $$G({n}_{i},\,v)={\int }_{{{\rm{\Omega }}}_{s}}{D}_{i}(\nabla {n}_{i}\nabla v+{k}_{B}T{q}_{i}{n}_{i}\nabla u\nabla v)dx,$$

10 $$Q({n}_{i},\,{v}_{s})=\sum _{N}{\int }_{N}(\nabla \cdot {D}_{i}(\nabla {n}_{i}+{k}_{B}T{q}_{i}{n}_{i}\nabla u)\cdot {v}_{s}dx\mathrm{.}$$

and the test functions *v*_*s*_ is

11 $${v}_{s}={\sigma }_{N}{{\bf{p}}}_{{\bf{i}}}\cdot \nabla v\mathrm{.}$$

We isolate the P*é*clet number Pe with a stability parameter of the form^60^

12 $${\sigma }_{K}=\frac{{h}_{N}}{2{\Vert {{\bf{p}}}_{{\bf{i}}}\Vert }_{2}}\xi ({{\rm{Pe}}}_{N}),$$

13 $${{\bf{p}}}_{{\bf{i}}}=-\,{D}_{i}{k}_{B}T{q}_{i}\nabla u$$

where *h*_*N*_ denotes the diameter of the element N, and

14 $${{\rm{Pe}}}_{N}=\frac{{\Vert {{\bf{p}}}_{{\bf{i}}}\Vert }_{2}{h}_{N}}{6{D}_{i}},$$

15 $$\xi ({{\rm{Pe}}}_{N})=\{\begin{array}{ll}{{\rm{Pe}}}_{N}, & 0\le {{\rm{Pe}}}_{N}\le \mathrm{1,}\\ \mathrm{1,} & {{\rm{Pe}}}_{N}\ge \mathrm{1,}\end{array}$$

where Pe_*N*_ denotes the P*e*′clet number of element N, which is an indication of the strength of advection. Poisson equations were solved using the Galerkin finite element method. More details and discussion can be found in the ref.^37^.

To make the iterations between the NP and Poisson equations to converge, the under-relaxation scheme was applied to solve the whole equtaions. In the process for solving equations, variables are updated with a linear combination of old values and calculated new values, rather than just using the new values. The under-relaxation scheme^61^ is described by

$${\tilde{u}}^{n}=t{u}^{n-1}+\mathrm{(1}-t){u}^{n}$$

$${{\tilde{n}}_{i}}^{n}=t{{n}_{i}}^{n-1}+\mathrm{(1}-t){{n}_{i}}^{n},$$

where the relaxation parameter 0 < *t* < 1 is a predefined constant and in this work, we set *t* as 0.8 for all computations.
